# Glycine-induced activation of GPR158 increases the intrinsic excitability of medium spiny neurons in the nucleus accumbens

**DOI:** 10.1007/s00018-024-05260-w

**Published:** 2024-06-17

**Authors:** Giuseppe Aceto, Luca Nardella, Simona Nanni, Valeria Pecci, Alessia Bertozzi, Sofia Nutarelli, Maria Teresa Viscomi, Claudia Colussi, Marcello D’Ascenzo, Claudio Grassi

**Affiliations:** 1 Fondazione Policlinico Universitario Agostino Gemelli, IRCCS, Rome, 00168 Italy; 2 Department of Neuroscience, Università Cattolica del Sacro Cuore, Rome, 00168 Italy; 3 Istituto di Analisi dei Sistemi ed Informatica “Antonio Ruberti”, National Research Council, Rome, Italy; 4 Department of Translational Medicine and Surgery, Università Cattolica del Sacro Cuore, Rome, 00168 Italy; 5 Department of Life Science and Public Health, Università Cattolica del Sacro Cuore, Rome, 00168 Italy

**Keywords:** GPR158, Glycine, Metabotropic glycine receptor, Intrinsic excitability, Medium spiny neurons, M-current

## Abstract

It has been recently established that GPR158, a class C orphan G protein-coupled receptor, serves as a metabotropic glycine receptor. GPR158 is highly expressed in the nucleus accumbens (NAc), a major input structure of the basal ganglia that integrates information from cortical and subcortical structures to mediate goal-directed behaviors. However, whether glycine modulates neuronal activity in the NAc through GPR158 activation has not been investigated yet. Using whole-cell patch-clamp recordings, we found that glycine-dependent activation of GPR158 increased the firing rate of NAc medium spiny neurons (MSNs) while it failed to significantly affect the excitability of cholinergic interneurons (CIN). In MSNs GPR158 activation reduced the latency to fire, increased the action potential half-width, and reduced action potential afterhyperpolarization, effects that are all consistent with negative modulation of potassium M-currents, that in the central nervous system are mainly carried out by Kv7/KCNQ-channels. Indeed, we found that the GPR158-induced increase in MSN excitability was associated with decreased M-current amplitude, and selective pharmacological inhibition of the M-current mimicked and occluded the effects of GPR158 activation. In addition, when the protein kinase A (PKA) or extracellular signal-regulated kinase (ERK) signaling was pharmacologically blocked, modulation of MSN excitability by GPR158 activation was suppressed. Moreover, GPR158 activation increased the phosphorylation of ERK and Kv7.2 serine residues. Collectively, our findings suggest that GPR158/PKA/ERK signaling controls MSN excitability via Kv7.2 modulation. Glycine-dependent activation of GPR158 may significantly affect MSN firing in vivo, thus potentially mediating specific aspects of goal-induced behaviors.

**Supplementary Information:**

The online version contains supplementary material available at 10.1007/s00018-024-05260-w.

## Introduction

Glycine acts as an inhibitory neurotransmitter in the spinal cord, brainstem, and cerebellum where it binds ionotropic glycinergic receptors that are permeable to chloride ions [1, 2]. Through these inhibitory receptors glycine participates in the control of fluxes of sensory information between the periphery and the CNS, as well as diverse motor activities like locomotion and respiration [3–6]. Glycine is also a co-agonist of N-methyl-d-aspartate glutamate receptors (NMDAR). Indeed, ion flux through these receptors depends on their interaction with both glutamate and glycine, the latter binding the GluN1 subunit [7].

Alongside activating ionotropic receptors, a recent seminal work has shown that glycine can also bind GPR158, an orphan Class C of G-protein-coupled receptor (GPCR) [8]. The discovery that GPR158 serves as a metabotropic glycine receptor (mGlyR) opened new perspectives on the understanding of the complexity of glycinergic transmission in the CNS, also considering that glycine and GPR158 have been implicated in a number of pathological conditions including depression [9–14].

GPR158 transduces signals by its interaction with the RGS7–Gβ5 complex, comprising the intracellular regulator of G-protein signaling 7 (RGS7) in complex with the G-protein beta-5 subunit (Gβ5) [8, 15–17]. Since RGS7-Gb5 is a selective guanosine triphosphatase (GTPase)-activating protein (GAP) for the inhibitory Gi/o proteins, which regulate cAMP production, inhibition of RGS7-Gb5 activity via GPR158 increases cAMP levels in neurons [8, 13]. Indeed, GPR158 activation produces an excitatory effect in many neuronal types by modulating key kinases, neurotrophic factors, and ion channels involved in neuronal excitability and synaptic transmission [14, 18–20].

GPR158 is highly expressed in brain regions implicated in the regulation of depression-related behaviors, such as the hippocampus, prefrontal cortex and nucleus accumbens (NAc) [14, 15, 21, 22]. However, little is known about neuronal adaptations following GPR158 activation in these brain areas, especially in the NAc, the main component of the ventral striatum that mediates goal-directed behaviors by integrating information from the neocortical, allocortical, thalamic, midbrain and brainstem structures [23, 24]. The major projection neurons that regulate goal-directed behaviors, the medium spiny neurons (MSNs; about 95% of total NAc neurons), are GABAergic neurons that can be distinguished in two subpopulations based on expression of dopamine receptors: D1R-MSNs expressing D1 receptors (D1R) and D2R-MSNs expressing D2 receptors (D2R) [25–27]. Alongside MSNs, cholinergic interneurons (CINs), comprising 1–3% of striatal neurons [28], are known to be critical for NAc functions [29, 30]. The high levels of GPR158 expression in the NAc and GPR158’s emerging role as an active modulator in physiological and pathological conditions prompted us to investigate the effects of GPR158 activation on intrinsic excitability of NAc MSNs and CINs. We found that GPR158 activation enhances intrinsic excitability in MSNs, but not in CINs, through PKA/ERK-dependent modulation of M-type potassium currents carried by Kv7 channels.

## Materials and methods

### Animals and ethical approval

We used male C57BL/6J mice. Colonies were established in the animal facilities at Università Cattolica del Sacro Cuore. The housing conditions were controlled, maintaining stable hygrometric and thermic conditions (50%; 21 °C ± 1 °C) on a 12-hour light/dark cycle with ad libitum access to food and water. Four- to six‐week‐old mice were used. All procedures complied with the Italian Ministry of Health (Law 116/92) and European Community Council (Directive 86/609/EEC) guidelines. This project has received approval from the Italian Ministry of Health (authorization n° 623/2022-PR).

### Slice preparation and electrophysiology

Coronal slices (300 μm) containing the nucleus accumbens were prepared as previously described [31–33]. C57/BL/6J mice postnatal days (P) 28–42 were anesthetized with isoflurane and killed by cervical dislocation and decapitated. The brains were quickly removed and placed in an ice-cold, sucrose-based cutting solution containing the following (in mM): TRIS-HCl 72, TRIZMA base 18, NaH_2_PO_4_ 1.2, NaHCO_3_ 30, KCl 2.5, glucose 25, HEPES 20, MgSO_4_ 10, Na-pyruvate 3, ascorbic acid 5, CaCl_2_ 0.5, sucrose 20. NAc slices (300 μm thick) were cut on a vibratome (VT1200S; Leica Microsystems, Germany) and soon transferred to an incubation chamber held at 32 °C and filled with a recovery solution containing (in mM): TRIS-HCl 72, HEPES 20, NaH_2_PO_4_ 1.2, TRIZMA base 18, KCl 2.5, NaHCO_3_ 25, CaCl_2_ 0.5, glucose 25, MgSO_4_ 10, Na-pyruvate 3, ascorbic acid 5, sucrose 20. After 30 min, slices were transferred to a second incubation chamber held at 32 °C and filled with artificial cerebrospinal fluid (aCSF) containing the following (in mM): NaCl 124, KCl 3.2, NaH_2_PO_4_ 1.2, MgCl_2_ 1, CaCl_2_ 2, NaHCO_3_ 26, and glucose 10, pH 7.4. During incubations, the chambers were continuously bubbled with 95% O_2_/5% CO_2_. Finally, slices were equilibrated at RT for at least 30 min. For electrophysiological recordings, slices were transferred to a submerged recording chamber constantly perfused at 2 ml/min with heated aCSF in the presence of the following synaptic blockers: picrotoxin (100 mM), strychnine (1 mM), NBQX (2,3-Dioxo-6-nitro-1,2,3,4-tetrahydrobenzo[f]quinoxaline-7-sulfonamide; 20 mM), and APV (D, L-2-amino-5-phosphonovaleric acid; 50 mM).

The MSNs of the NAc were visualized under DIC infrared illumination. Patch pipettes had a resistance of 4–6 MΩ when filled with an internal solution containing (in mM): K-gluconate 145, MgCl_2_ 2, HEPES 10, EGTA 0.1, Na-ATP 2.5, Na-GTP 0.25, phosphocreatine 5, pH adjusted to 7.2 with KOH. NAc cholinergic interneurons were selected for recordings based on their characteristic somatodendritic morphology, and their identity was confirmed by their stereotyped responses to current injection such us depolarizing sag that developed during a hyperpolarizing step of current, a rebound depolarization observed after the end of a hyperpolarizing step, and long-lasting afterpolarizations after bursts of spikes [34]. Recordings were performed using a Multiclamp 700B/Digidata 1550 A system (Molecular Devices, USA) and digitized at a 10,000 Hz sampling frequency. All the electrophysiological recordings were analyzed using the Clampfit 10.9 software (Molecular Devices). Evoked firings were recorded in whole-cell, current-clamp mode. Only cells with a stable resting membrane potential negative to − 70 mV, overshooting action potentials (exceeding 70–80 mV threshold-to-peak), and an input resistance > 100 MΩ were included. Additionally, cells were rejected if there was a 20% change in the input resistance and resting membrane potential. The membrane input resistance was measured by a series of 600 ms hyperpolarizing current steps from − 50 to 0 pA, step 10 pA with 1 s interval.

To test the firing properties of MSNs during continuous depolarization, we exposed these cells to a series of 1 s of somatic current pulses (every 10 s). The minimal amplitudes needed to cause a steady firing of 4–8 action potentials were used to determine the current levels. Beginning with 80 pA, the injected current was gradually increased in 10–50 pA increments until these criteria were satisfied. Of note, during interpulse intervals, the membrane potentials were maintained at resting value (∼80 mV for MSNs and ∼60 mV for CINs) by injecting hyperpolarizing/depolarizing currents. All the electrophysiological parameters were analyzed in 30–60 s (pre) and 5–10 min (post) drug application.

Measurements of action potential half-width and all action potential properties were taken from waveform averages representing 1 s per condition. AHP voltages were measured individually from raw traces before averaging.

The Kv7 currents were recorded in voltage–clamp mode. Currents induced by voltage steps from − 10 to − 60 mV preceded by a 4 s prepulse to 0 mV. Membrane capacitances and series resistance (Rs) were compensated electronically. Before compensation, Rs ranged from 5 to 20 MΩ and were regularly corrected by 75 to 85%. Data obtained from a given cells were rejected if Rs were larger than 20 MΩ or changed by > 20% during the experiment. The current amplitude was measured between the peak and the end of the voltage step. Potentials were not corrected for the liquid junction potential. Analysis and curve fitting were conducted in Clampfit and SigmaPlot 14.0 (Systat Software Inc., San Jose, CA, USA). The peak conductance (G_p_) was calculated as G_p_ = Ip/(V_c_ − V_rev_), where I_p_ is the peak current, V_c_ is the command voltage and V_rev_ is the estimated reversal potential for K^+^ (− 95 mV). The G_p_–V relationships were described by assuming a first-order Boltzmann function: G_p_(V) = G_pmax1_/(1 + exp ((V_c_ − V_1/2_)/k), where G_pmax_ is the maximal peak conductance, k is the slope factor and V_1/2_ is the activation midpoint voltage.

To investigate the effects of GPR158 activation, 1 mM of Glycine was used based on the assumption that this concentration ensures maximal receptor stimulation in ex vivo NAc slices as in a recent study by Laboute and colleagues [8].

XE991-sensitive currents were obtained by subtracting the traces in the presence of XE991 (20 µM; the selective blocker of Kv7 channels), from those obtained before drug administration (upper left trances). Current densities were obtained dividing Kv7/M current amplitude by membrane capacitance. Membrane capacitance was calculated using the equation: capacitance = membrane time constant/input resistance [35].

### Multiplex RNAscope™ and immunofluorescence and confocal analysis

RNAscope™ (Advanced Cell Diagnostic, USA) was performed according to the manufacturer’s protocol as in Wang and colleagues [36]. Briefly, adult C57BL/6J mice were deeply anesthetized, and the brains were collected. Afterward, brains were overnight post-fixed in 4% PFA and then were cryoprotected in sucrose solution (30%). 15 μm coronal slices were sectioned using a cryostat and mounted on Superfrost Plus slides. Sections were dehydrated and post-fixated according to the protocol, then peroxidase was applied, and antigen retrieval was performed. The following RNA probes were used: Mm-Gpr158-C1 (#524,851); Mm-Drd1 (#461,901-C2) and Mm-Drd2 (#406,501-C3) respectively for Gpr158, D1R and D2R was applied on sections. Then the amplification cascade was performed as stated in the manufacturer’s protocol and detected by using TSA-Vivid™ fluorophores 570 and 520. Before RNAscope processing, sections were incubated with primary antibodies including rabbit anti-Kv7.2 antibody (1:200; cod. D9L5S; Cell signaling), goat anti-ChAT (1:200; Cod. #AB144P; Sigma- Aldrich), and rabbit anti-GRP158 (1:200; cod. #PA5-102092; Thermo Fischer Scientific) in 0.1 M PB. After the RNAscope protocol, sections were incubated for 1 h at RT with secondary antibodies including Alexa Fluor 647 donkey anti-rabbit IgG or Alexa Fluor 647 donkey anti-goat (1: 200; Molecular Probes). Sections were air-dried and coverslipped with GEL/MOUNT (Biomeda, Foster City, CA, USA). Sections were examined under a confocal laser-scanning microscope Nikon APM1 equipped with four laser lines: violet diode emitting at 405 nm (for DAPI), argon emitting at 488 nm, and helium/neon emitting at 543 nm and 633.

Quantitative analysis of Gpr158-mRNA, Kv7.2 protein, GPR158 protein, and Gpr158-mRNA/GPR158 protein co-localization was performed offline on confocal images acquired through the 20× objective at the 0.01 zoom factor. All double-labeled cells in four sections, regularly spaced throughout the caudo-rostral extent of the NAc, were counted. The Gpr158-mRNA, Kv7.2 protein, and GPR158 protein immunofluorescence (optical density O.D.) was performed by densitometric analyses. Specifically, GPR158-mRNA, Kv7.2 protein, and GPR158 protein-associated signals were quantified by manually outlining individual cells and measuring cell-associated fluorescence intensity with the ImageJ software (http://rsb.info.nih.gov/ij/). The F/A ratio defines the mean fluorescence of individual cells (F) normalized to the total cellular surface (A). Quantification was done on 50 cells per mice for MSNs and on 15 cells for CINs (*n* = 4 mice).

Cellular co-localizations between Gpr158-mRNA, and GPR158 protein on neurons were analyzed by counting and characterizing cell labeling off-line through the Nikon proprietary image analysis program. Two digital images of the same optical section (one for each laser channel, green/or blue and red) were acquired and digitally merged in a third image, which was used for cell counting. The features of immunolabeled cells were analyzed by zooming on the cells and by serially excluding each channel (green and red) to better appreciate the cellular morphology. Double and single immunolabeled cells were then digitally marked, and recorded, and the material stored in a data archive.

### Western-blot assay

Western blot experiments were performed as previously described [37]. Briefly, tissues were lysed in ice-cold lysis buffer (NaCl 150 mM, Tris-HCl 50 mM pH 7.4, EDTA 2 mM) containing 1% Triton X-100, 0.1% SDS, 1 × protease inhibitor cocktail (Sigma-Aldrich, St Louis, MO, USA), 1 mM sodiumorthovanadate (Sigma-Aldrich) and 1 mM sodium fluoride (Sigma-Aldrich). Lysates were incubated for 10 min on ice with occasional vortexing and spun down at 22,000 g at 4 °C. The supernatant was quantified for protein content (DC Protein Assay; Bio-Rad, Hercules, CA, USA). Equal amounts of protein were diluted in Laemli buffer, boiled, and resolved by SDS-PAGE. Primary antibodies for anti-ERK1/2 (WB 1:1000, monoclonal, rabbit cell signaling #9102S), anti-pERK1/2 (WB 1:1000, polyclonal, rabbit cell signaling #4370S), anti-Actine (WB 1:2000 mouse Sigma Aldrich A2228) were incubated overnight and revealed with HRP-conjugated secondary antibodies (Cell Signaling Technology). Expression was evaluated and documented by using UVItec Cambridge Alliance. The band of interest was normalized to the housekeeping (Actine) and expressed as fold change versus control. Images shown were cropped for presentation with no manipulations.

### Immunoprecipitation

Evaluation of Kv7.2 total phosphorylation level was performed by immunoprecipitating the specific protein (Kv7.2) and analyzing the level of phosphorylation on serine residues (anti-phospho-serine Abcam 1:1000 ab9332) by western blot. The ratio of Kv7.2 and phospho-serine signals was used as index of Kv7.2 serine phosphorylation. Immunoprecipitation was performed using 2 µg of antibody for 200 µg of protein extract with the Ademtech´s Bio-Adembeads paramagnetic bead system. Negative controls were performed with the same amount of protein extract sample immunoprecipitated with the corresponding purified IgG (Santa Cruz). Total lysates were run in the WB as loading controls.

### Droplet digital PCR analysis

Analysis by ddPCR on single MSN or CIN was performed as previously described [38]. Briefly, intracellular contents, drawn into the tip of the patch pipette by applying negative pressure, were then transferred to RNase/DNase-free tubes as described in Cadwell and colleagues [39] and processed as in previous works [38, 40]. Four microlitres preAmp (1:10 dilution) were used to perform ddPCR in duplicate with EVA green to obtain a total droplet number > 14 000. Data are quantified as copy number µl mRNA levels were normalized to housekeeping gene GAPDH. The data in Figs. 2M and N and 8M and N are represented as box plots showing the median, upper, and lower quartiles of the data and minimum and maximum values as whiskers. Primers for GAPDH were as in Aceto et al [38]. Primers are as follows: DRD1 5’-AGATCGGGCATTTGGAGAGAT-3’ and 5’-TGCTGCCTCTTCTTCTGAGACA-3’; DRD2 5’-TCTGGAGGTGGTGGGTGAGT-3’ and 5’-TGCTTGCTGTGCACATCATG-3’; GPR158 5’-CTGCCACCTTAACAACTCAGAGTGT-3’ and 5’-CTGATAGGCTCCAAGCACGAA-3’; ChAT 5’-AGCTTGAATGGAGCGAATCG-3’ and 5’-TCTCGGCCCACCACAAAC-3’.

### Statistical analysis

Data are expressed as means ± SEM. Statistical significance was assessed with either Student’s *t-*test or one-factor ANOVA for multiple-group comparisons (with Bonferroni post hoc test). Statistical analysis was performed with the SYSTAT 10.2 software (Systat Software). Two-tailed, non-parametric statistical comparisons were performed using the Wilcoxon signed-rank (WSR) test for paired data or the Mann-Whitney U (MWU) test for unpaired data. The level of significance was set at 0.05. n = number of individual cell recorded, each cell from a new slice, and each experiment from ≥ 3 mice for all electrophysiology experiments with no more than three cells from one any animal per experiment.

## Results

### GPR158 activation enhances the evoked firing of medium spiny neurons through a PKA-dependent mechanism

We first sought to determine the effects of GPR158 activation on MSN excitability. To this aim, we performed whole-cell patch-clamp recordings of identified NAc MSNs in *ex vivo* brain slices during perfusion of glycine (1 mM), the newly discovered agonist of GPR158 [8]. MSNs, which represent > 95% of neurons in the NAc, were identified by their electrophysiological characteristics: ramp depolarization at subthreshold levels, regular AP firing when stimulated with over-threshold voltages, and hyperpolarized resting membrane potential [41, 42].

**Fig. 1:**
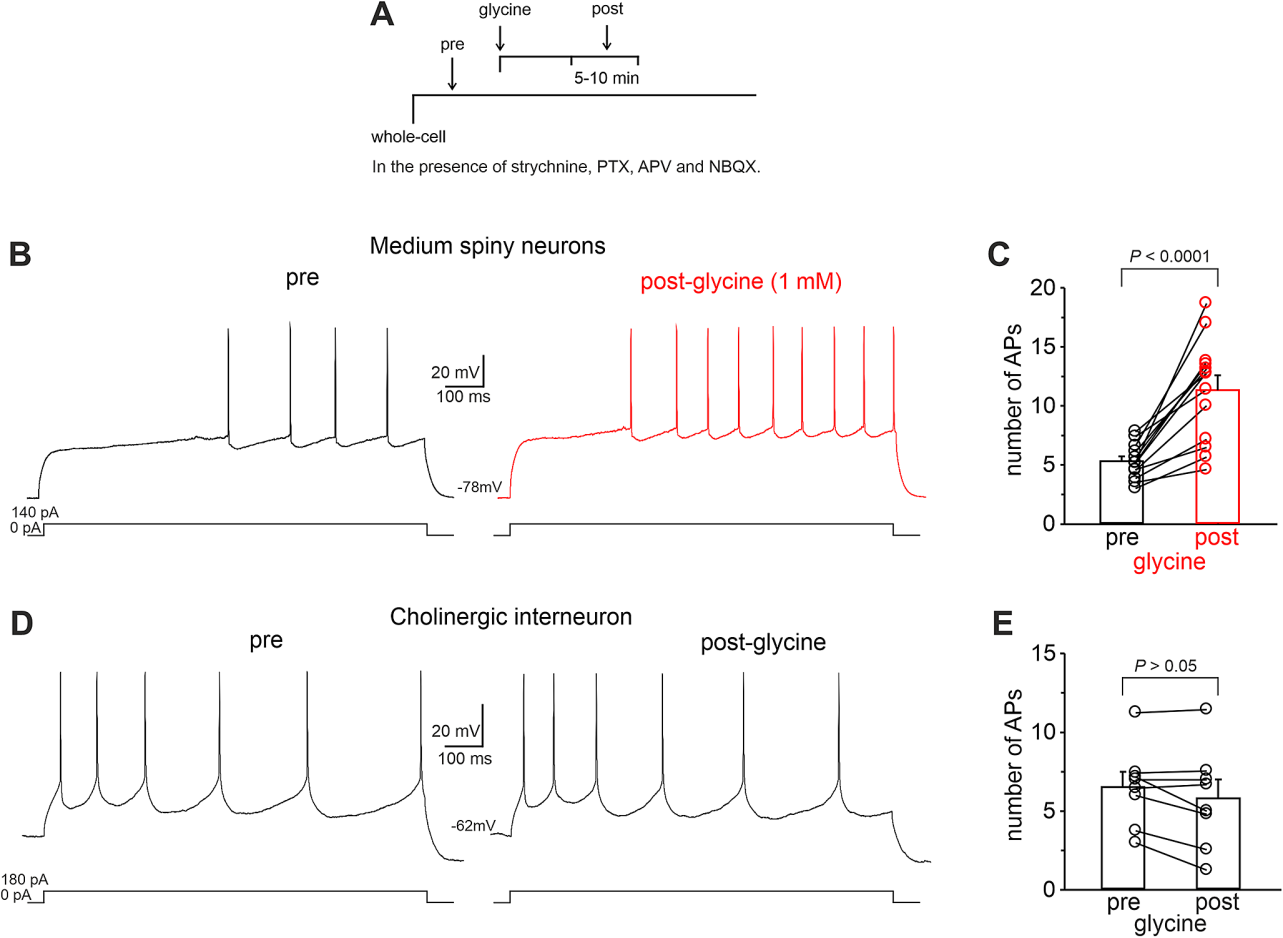
Glycine increases evoked firing frequency in MSNs through GPR158 activation. **A** Experimental protocol: following the establishment of the whole-cell configuration in current-clamp mode, the number of action potentials was measured (pre). The AP number was also measured following 5–10 min of continuous perfusion with 1 mM glycine (post). Experiments were conducted in the presence of strychnine (1 µM; blocking glycine and acetylcholine receptors), picrotoxin (100 µM; blocking GABA receptors), APV (D, L-amino-5-phosphonovaleric acid; 50 µM; blocking NMDA receptors) and NBQX (2,3-Dioxo-6-nitro-1,2,3,4-tetrahydrobenzo[f]quinoxaline-7-sulfonamide; 20 µM blocking AMPA receptors) to isolate the metabotropic action of glycine. **B** Representative traces showing current-evoked firing of an MSN before (black) and after (red) perfusion of glycine (1mM). The current-evoked firing protocol is depicted on the bottom. **C** Normalized mean ± S.D. for 13 experiments from 7 mice in which glycine was applied (*P* < 0.0001; paired Student’s *t*-test). **D** Representative traces and summary plot **E** illustrating that glycine application did not affect evoked firing in CINs (*n* = 8 from 3 mice; *P* > 0.05; paired Student’s *t*-test)

To test the firing properties of MSNs during long-lasting depolarization, we applied 1-s somatic current pulses whose levels were chosen, in each recorded MSN, to elicit a stable firing of 4–8 action potentials. Glycine was bath applied for 5–10 min (Fig. 1A) in the presence of picrotoxin (100 µM), strychnine (1 µM), NBQX (2,3-Dioxo-6-nitro-1,2,3,4-tetrahydrobenzo[f]quinoxaline-7-sulfonamide; 20 µM), and APV (D, L-2-amino-5-phosphonovaleric acid; 50 µM) to block its action on ionotropic receptors. Under these experimental conditions, application of glycine significantly increased the evoked firing in NAc MSNs (number of APs: pre = 5.26 ± 0.41; post = 11.29 ± 1.24; n *=* 13 cells from 5 mice; *P* < 0.0001; paired Student’s *t-*test; Fig. 1B, C), an effect that was absent when ACSF (vehicle) was applied (number of APs: pre = 5.56 ± 0.62; post = 5.47 ± 0.76; *n* = 13 cells from 5 mice; *P* > 0.05; paired Student’s *t-*test; Supplementary Fig. 1A, B). Of note, glycine’s effect on evoked firing was independent of MSN localization in NAc core and shell (Supplementary Fig. 1C; NAc shell; number of APs: pre = 5.79 ± 0.63; post = 11.8 ± 1.5; *n* = 7 cells from 3 mice; *P* < 0.05; paired Student’s *t-*test; NAc core; number of APs: pre = 4.64 ± 0.48; post = 10.80 ± 2.31; *n* = 6 cells from 2 mice; *P* > 0.018; paired Student’s *t-*test). In a subset of experiments, we observed that twenty minutes of washout from glycine induced a significant reduction of the evoked firing toward the control values, although the MSNs retained some degree of hyperexcitability (number of APs: pre = 6.69 ± 0.93; post = 11.17 ± 1.3; wash = 8.67 ± 0.84; *n* = 6 cells from 2 mice; *P* < 0.05; paired Student’s *t-*test).

We then tested whether glycine application could also affect the evoked firing of cholinergic interneurons (CINs). NAc CINs were identified based on their very large somata [43] and their distinctive electrophysiological properties, which included: *i*) a depolarizing sag that developed during a hyperpolarizing step of current, *ii*) a rebound depolarization observed after the end of a hyperpolarizing step, and (*iii*) marked, long-lasting afterpolarizations after bursts of spikes [43–45]. Interestingly, glycine application did not produce any changes in the intrinsic excitability of CINs (Fig. 1D, E and Supplementary Fig. 1D; number of APs: pre = 6.51 ± 0.95; post = 5.79 ± 1.19; n *=* 8 cells from 3 mice; *P* > 0.05; paired Student’s *t-*test) suggesting that GPR158 may be differently expressed in MSNs and CINs.

**Fig. 2:**
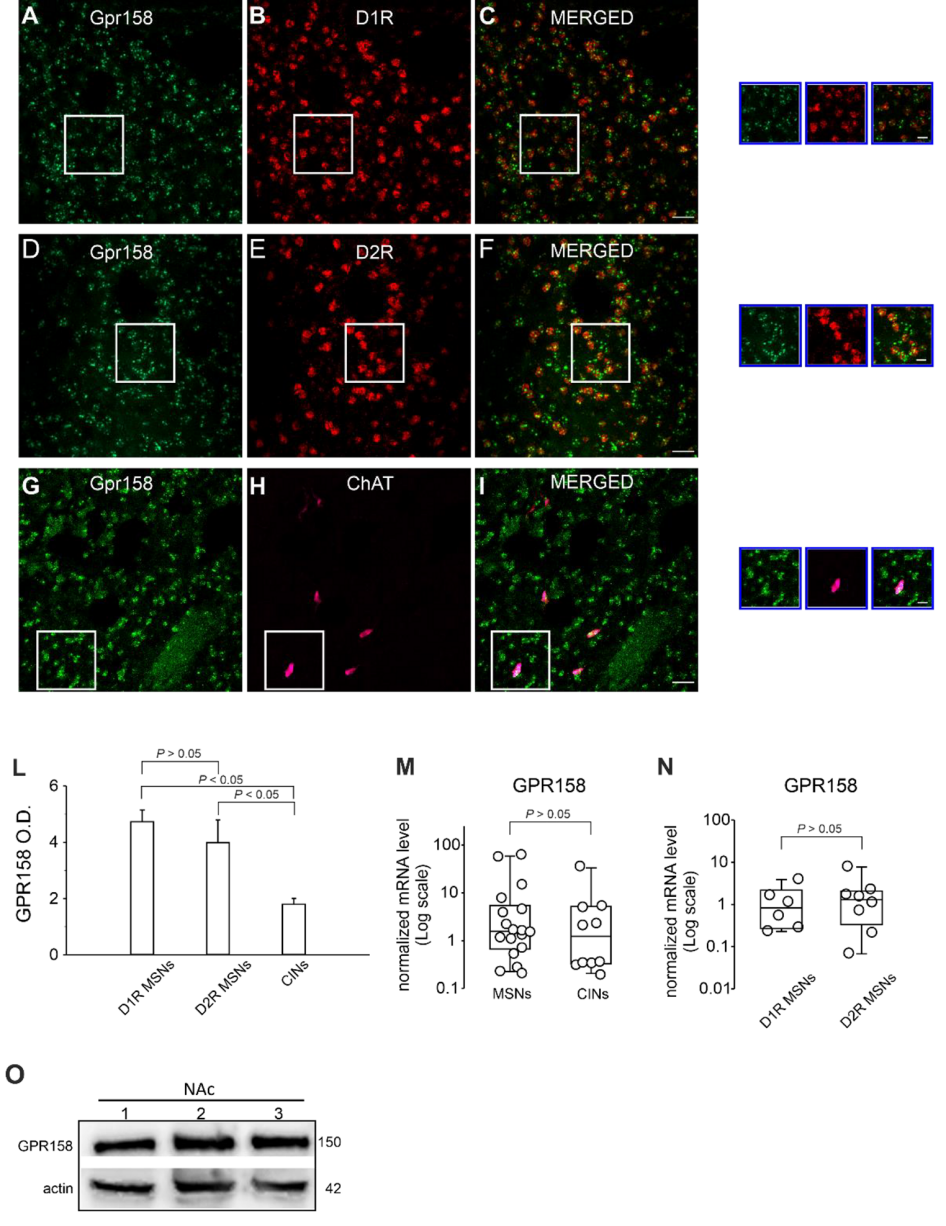
Expression of GPR158 in the NAc MSNs expressing D1R or D2R and in CINs. **A**, **C** Representative multiplex RNAscope and immunofluorescence images illustrating high density of Gpr158 mRNA signals in NAc D1 MSNs. **D**, **F** High-density Gpr158 mRNA was also detected in D2 MSNs. **G**, **I** Gpr158 mRNA signal in CINs was significantly lower than in MSNs as shown in panel **L** summarizing averages of GPR158 optical densities in single MSNs expressing either D1R or D2R, and CINs. Quantification was done on 50 cells per mice for MSNs and on 15 cells for CINs (*n* = 4 mice). Scale bars = 25 μm; insets 10 μm. **M** Summary plot of Gpr158 mRNA expression assessed by droplets digital PCR in single MSNs (*n* = 18 from 6 mice) and CINs (*n* = 10 from 4 mice). **N** Summary plot showing quantification of Gpr158 mRNA signal in single MSNs expressing either D1Rs (*n* = 6 from 2 mice) or D2Rs (*n* = 8 from 3 mice). **O** Representative western blots of NAc tissues showing GPR158 protein levels

To test this hypothesis, we first employed RNAscope fluorescent in situ hybridization (FISH) combined with immunofluorescence (IF) in fixed CNS tissue sections which allows reliable quantification of multiplexed transcript detection within single IF-labeled cells. Tissue sections containing the NAc were used for multiplexed hybridization using distinct mRNA probes for Gpr158, D1R, and D2R combined with IF for choline acetyltransferase (ChAT) to identify CINs. As shown in Fig. 2, Gpr158 mRNA expression levels were comparable in D1R and D2R MSNs (average optic densities: D1 = 4.73 ± 0.35; D2 = 3.98 ± 0.70; one-way ANOVA; F _(2,9)_ = 2.58; *P* > 0.05; followed by Bonferroni *post hoc* test) suggesting that the GPR158 signaling pathway may affect intrinsic excitability in both types of MSNs. Interestingly, and in line with our electrophysiology findings, lower Gpr158 mRNA were found in CINs compared to MSNs (Fig. 2; Average optic density: CINs = 1.79 ± 0.19; one-way ANOVA; F_(2,9)_ = 2.58; *P* < 0.05; followed by Bonferroni *post hoc* test). Accordingly, experiments in which the mRNA probes for Gpr158 were coupled with GPR158 antibody also showed lower levels of protein in CINs (Supplementary Fig. 2). To Gpr158 mRNA expression was further investigated is single MSNs and CINs by using a protocol that combines whole-cell patch-clamp recordings with high-quality single-cell RNA analysis by droplet digital PCR (single-cell qRT-ddPCR) [38, 40, 46]. We found that Gpr158 mRNA expression in single MSNs expressing either D1R or D2R MSNs was comparable, in line with the results of RNAscope experiments (Fig. 2N). The Gpr158 mRNA expression in CINs was lower though not significantly different from that of MSNs (Fig. 2M). Of note, these data indicated the total RNA levels contained in each cell and cell size of CINs, which are the biggest neurons in the neostriatum (even > 40 μm), is far greater than that of MSNs. As such, Gpr158 density in CINs is markedly lower than in MSNs. Finally, GPR158 protein levels in NAc tissues were also assessed by western blot analysis (Fig. 2O).

Collectively, these findings suggest that glycine elicits positive modulation of MSN excitability by activation of the GPR158-signaling pathway, an effect that was missing in CINs likely because of the lower receptor density.

**Fig. 3:**
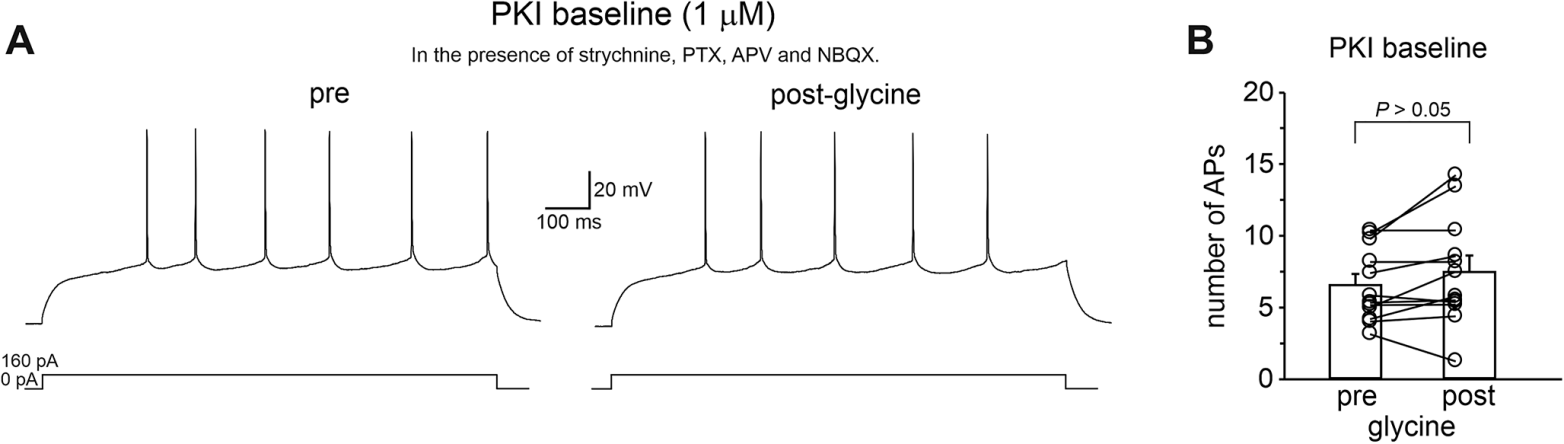
Glycine-dependent modulation of evoked firing requires a PKA signaling pathway. **A** Representative traces showing that slice incubation (5–10 min) with the PKA inhibitors PKI-14-22 amide, myristoylated (1µM) prevented the glycine-dependent modulation of evoked firing in MSNs. **B** Summary graph depicting quantification of evoked firing in the experimental conditions reported in panel A (*n* = 12 from 5 mice; *P* > 0.05; paired Student’s *t*-test)

It has been shown that inhibition of RGS7-Gb5 activity, via GPR158 activation, affects cAMP levels in neurons [8, 13]. Since the cAMP/protein kinase A (PKA) pathway is one of the prominent and well-characterized signaling pathways in MSNs affecting a variety of intracellular targets including ion channels that regulate MSN excitability [47–49], we hypothesized that PKA-mediated protein phosphorylation could be involved in GPR158-dependent increase in evoked firing we observed in MSNs. To test this hypothesis, we bath applied a cell-permeable peptide inhibitor of PKA, PKI 14–22 amide, myristoylated (PKI 1 µM), for at least 10 min before glycine application. In the presence of PKI, GPR158 activation failed to significantly affect MSN firing rates (Fig. 3; number of APs: pre = 6.54 ± 0.76; post = 7.47 ± 1.14; *n* = 12 cells from 5 mice; *P* > 0.05; paired Student’s *t*-test) indicating that positive modulation of MSN excitability by glycinergic transmission requires intact PKA signaling.

**Fig. 4:**
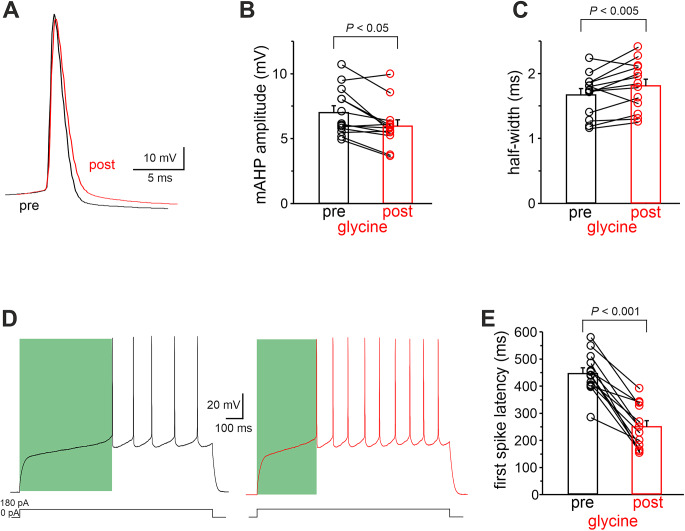
Glycine-dependent GPR158 activation affects mAHP amplitude, half-width, and first spike latency. **A** Superimposition of APs highlighting decreased mAHP amplitude and broadened AP following glycine application. **B**, **C** Summary plot showing mean values of mAHP and AP half-width before and after glycine application (*n* = 13 cells from 5 mice). **D** Representative traces showing reduced first spike latency following glycine perfusion. **E** Summary plot depicting quantification of the experiments shown in D (*n* = 13 cells from 5 mice)

We next sought to identify the molecular downstream target of PKA underlying the MSN intrinsic excitability modulation we observed following GPR158 activation. We first analyzed MSN AP properties before and after glycine application. As shown in Fig. 4A, B, we observed a significant reduction in mAHP after glycine application (mAHP amplitude: pre = 7.01 ± 0.53 mV; post: 5.97 ± 0.49 mV; n *=* 13 cells from 5 mice; *P* < 0.05; paired Student’s *t-*test). GPR158 activation also significantly increased the AP half-width (Fig. 4C; AP half-width: pre = 1.67 ± 0.09 ms; post = 1.81 ± 0.10 ms; n *=* 13 cells from 5 mice; *P* < 0.05; paired Student’s *t-*test). Moreover, we observed a significant reduction in the first spike latency (pre = 445.5 ± 21.7 ms; post = 249.0 ± 22.8 ms; n *=* 13 cells from 5 mice; *P* < 0.001; paired Student’s *t*-test; Fig. 7D, E). GPR158-dependent activation of the PKA signaling pathway may elevate MSN excitability through its effects on multiple classes of ion channels. However, the above changes in AP properties are consistent with negative modulation of K^+^ conductances, such as the M-currents, that in the central nervous system are mainly carried out by Kv7/KCNQ channels.

The Kv7/KCNQ/M channels are a subfamily of voltage-gated K^+^ channels with five members, Kv7.1 to Kv7.5, encoded by KCNQ1–5 genes [50–52]. In the brain, Kv7/M channels are formed by heterotetramers of the different subunits with Kv7.2/7.3 being the most prevalent [53–55]. Kv7/KCNQ/M channels regulate neuronal excitability by controlling repetitive firing, and the suppression of spontaneous firings [50]. Consistently with our findings, it has been shown that Kv7/KCNQ/M channel downregulation influences the amplitude of mAHP and the duration of AP [56–59].

### GPR158 stimulation increases evoked firing through reduced Kv7/M-mediated currents

**Fig. 5:**
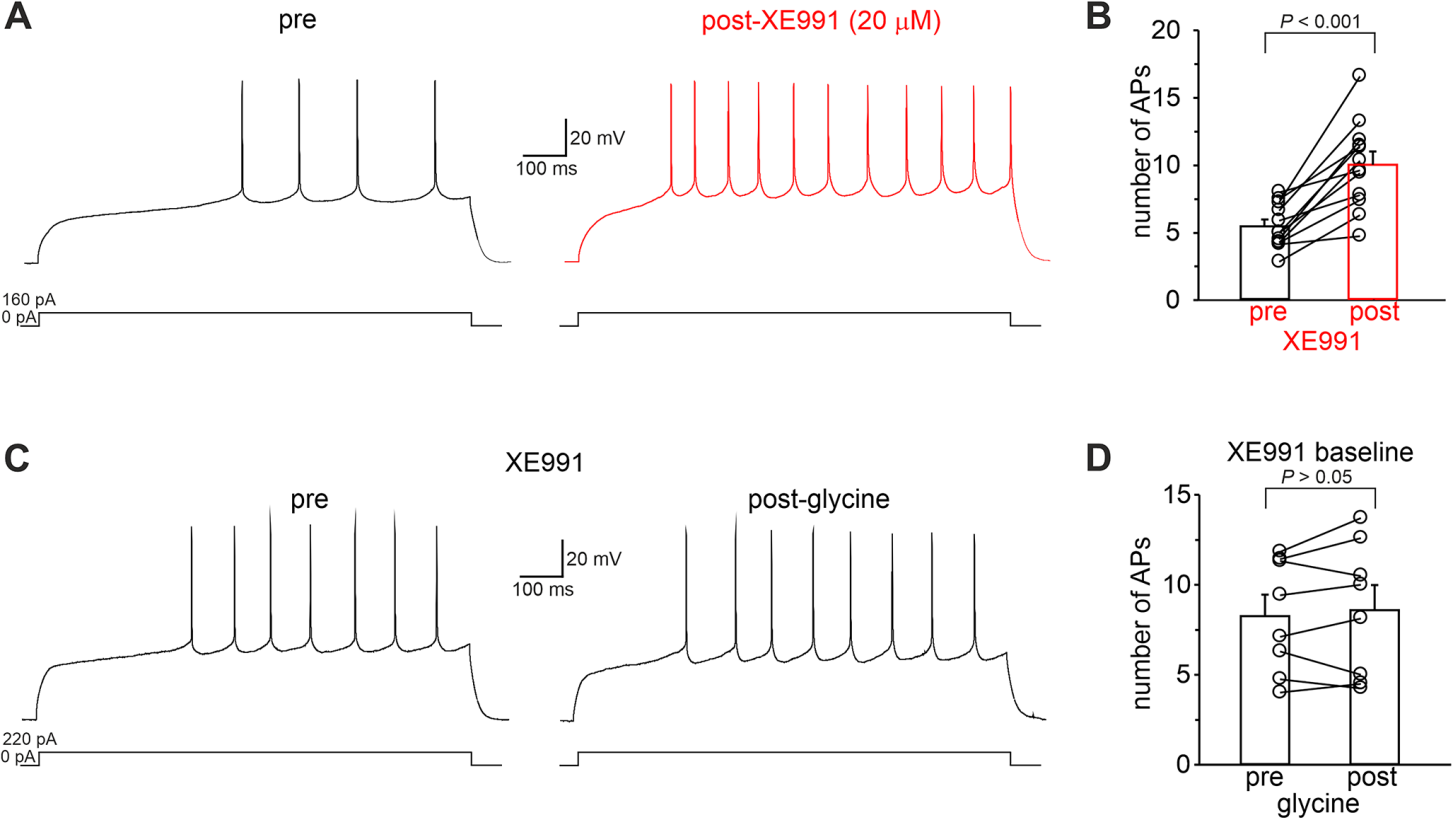
Pharmacological inhibition of Kv7 channels mimics and occludes glycine’s effect on evoked firing. **A** Representative traces showing current-evoked firing in an MSN before (black) and after (red) local perfusion of the KCNQ channel inhibitor XE991 (20 µM). **B** Bar graph showing mean AP number in which XE991 was applied (*n* = 12 cells from 5 mice; *P* < 0.001; paired Student’s *t*-test). **C** Representative traces showing that the presence of XE991 in the bath prevented the increase of the evoked firing induced by glycine application. **D** summary plot illustrating quantification of the evoked firing in the experimental conditions reported in C (*n* = 8 cells from 3 mice; *P* > 0.05; paired Student’s *t*-test)

Data shown so far suggest that negative modulation of Kv7/M channels may underlie GPR158-induced increase in evoked firing. To test this hypothesis, we performed a new set of experiments in which the effect of glycine on MSN-evoked firing was studied in the presence of the specific Kv7 channel blocker, XE991. We hypothesized that if the GPR158-dependent increase in evoked firing was due to reduced Kv7/M channel activity, the pharmacological blockade of these channels would attenuate the effect of glycine. We first examined the effects of the pharmacological Kv7/M current blockade on evoked firing. As shown in Fig. 5A, B, when 20 µM XE991 was applied, the firing was significantly increased (number of APs: pre = 5.44 ± 0.48; post = 10.01 ± 0.98; *n* = 12 cells from 5 mice; *P* < 0.001; paired Student’s *t-*test). Interestingly, the magnitude of the increased evoked firing by XE991 was similar to that observed when glycine was applied (Fig. 1B). Of note, XE991 also mimicked the glycine’s effects on first spike latency, mAHP and half-width (Supplementary Fig. 3; first spike latency: pre = 376.7 ± 43.0 ms; post = 189.1 ± 20.7 ms; *n* = 12 cells from 5 mice; *P* < 0.001; paired Student’s *t-*test; mAHP amplitude: pre = -7.98 ± 0.43 mV; post: -6.69 ± 0.28 mV; *n* = 12 cells from 5 mice; *P* < 0.05; paired Student’s *t-*test; AP half-width: pre = 1.37 ± 0.16 ms; post = 1.61 ± 0.24 ms; *n* = 12 cells from 5 mice; *P* < 0.05; paired Student’s *t-*test).

We then performed occlusion experiments by recording evoked firing in brain slices perfused with XE991 before and after glycine application. Under these experimental conditions, evoked firing was not significantly increased by GPR158 activation (Fig. 5C, D; number of APs: pre = 8.27 ± 1.9; post = 8.59 ± 1.40; *n* = 8 cells from 3 mice; *P* > 0.05; paired Student’s *t-*test). These findings supported our hypothesis that the increased firing following GPR158 activation relies on downregulation of Kv7/M channels.

**Fig. 6:**
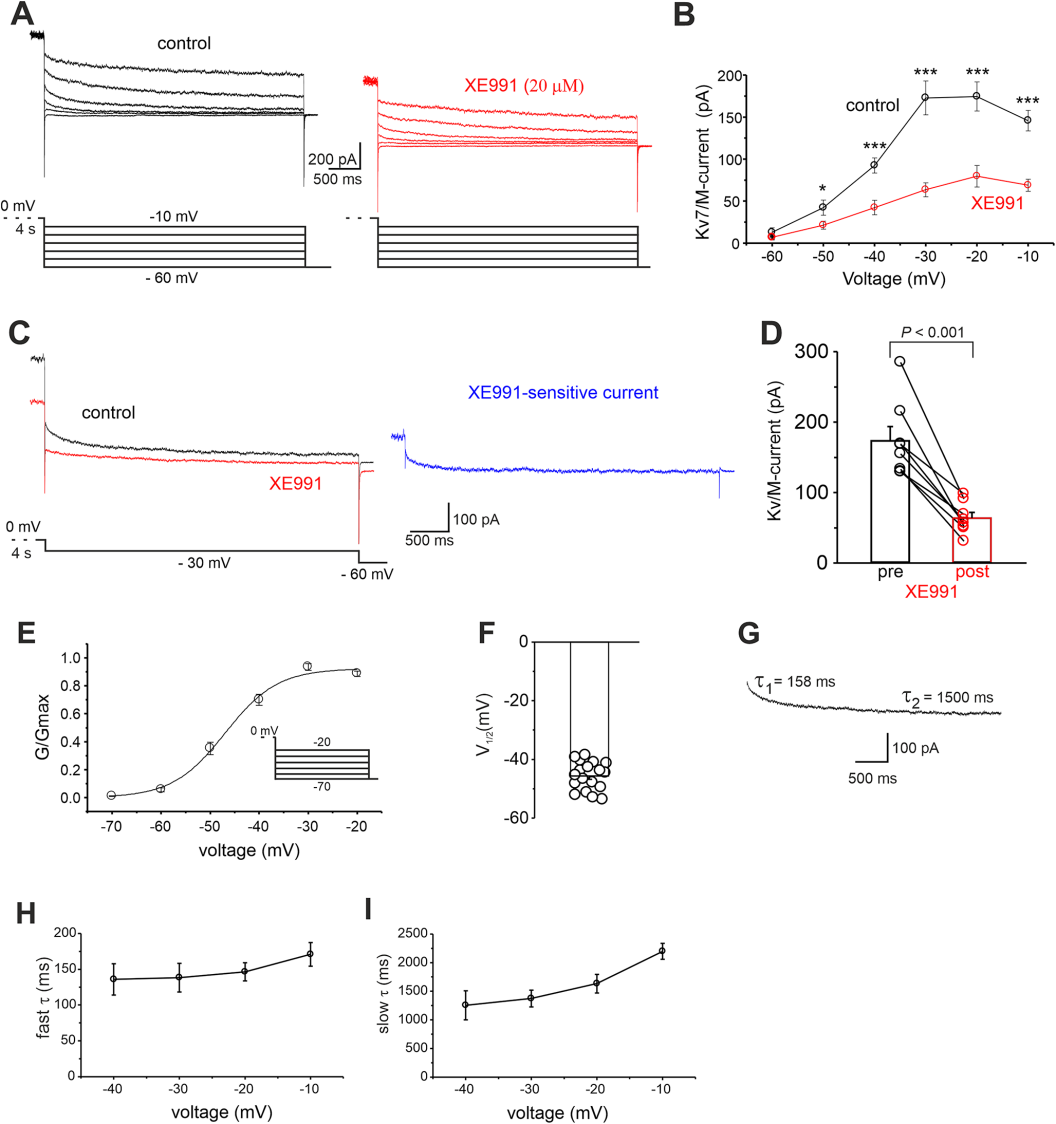
Isolation and characterization of Kv7/M-currents in NAc MSNs. **A** Voltage-clamp recordings of KCNQ currents in an MSN before and after bath application of the specific KCNQ blocker XE991 (20 µM). Currents were induced by voltage steps from − 10 to -60 mV preceded by a 4 s prepulse to 0 mV. **B** Current-voltage relationship for KCNQ current before and after XE991 application (*n* = 8 from 3 mice; *p < 0.05 paired Student’s *t*-test; ***p < 0.001 paired Student’s *t*-test). The current amplitudes were measured between the peak and after 1 s of the voltage step. **C** Representative traces showing KCNQ currents recorded at -30 mV before and after XE991 application. The XE991-sensitive current (right; blue trace) was obtained by subtracting the trace in the presence of XE991 (left; red trace) from that obtained before drug application (left; black trace). **D** Summary plot showing the decreased KCNQ currents by XE991 (*n* = 8 cells from 3 mice; *p* < 0.001; paired Student’s *t*-test. **E** Conductance-voltage relationship. G, conductance; Gmax, maximum G. Gmax was determined from the amplitude of the current relaxation during the hyperpolarized steps. The data points were fitted using the following equation: G_p_(V) = Gpmax1/(1 + exp ((Vc − V_1/2_)/k), where V_1/2_ is the midpoint voltage. (E, Insets) Protocols used to determine the conductance-voltage relationship. (**F**) Bar graph showing the mean ± SD of V_1/2_ (*n* = 19 from 6 mice). **G** Representative trace for a step from 0 mV holding potential to -30 mV, showing that the deactivation process had two-time constants. The deactivation time constants are shown next to the current trace. **H**, **I** Voltage dependence of fast and slow time constants

We next performed whole-cell patch-clamp recordings in voltage-clamp configuration and compared the magnitude of Kv7/M-current before and after GPR158 activation in MSNs. The Kv7/M currents are slowly activated and deactivated without inactivation. To measure these currents in relative isolation, a holding potential of 0 mV was used. At this potential, Kv7/M current is steadily activated, whereas most other voltage-gated ion channels are largely inactivated, thus eliminating Ca^2+^ currents, the A-type K^+^ currents, and delayed rectifier currents [60–64]. Negative-going steps (-10 mV) from a holding potential of 0 mV evoked a slow inward relaxation (Fig. 6A) characteristic of Kv7/M-current deactivation [61]. The voltage dependence of Kv7/M-currents deactivation was assessed by converting the current amplitudes to peak conductances. The peak conductance-voltage relationship for each cell was described by a first-order Boltzmann function with a deactivation midpoint (V_1/2_) of – 45.6 ± 1.1 mV (Fig. 6E, F; *n* = 19 from 6 mice). The deactivation kinetics of the Kv7/M-currents were biphasic over a range of potentials (Wang et al. 1998; Shen et al. 2005), which was also the case under our experimental conditions. The deactivation time constants at – 30 mV were 138.2 ± 20 ms and 1372.1 ± 145 ms (Fig. 6G, I). The gating properties of the recorded channels closely resemble those of the Kv7 family [51, 53, 56, 57], and biophysical characterization was further supported by pharmacology. Indeed these outward M-currents were inhibited by XE991, a selective Kv7 channel blocker (Fig. 6; pre = 169.9 ± 37.5.4 pA; post = 65.4 ± 14.2; *n* = 7 cells from 3 mice; *P* < 0.05; paired Student’s *t-*test) [50], indicating that slow decay currents were indeed mediated by Kv7/M channels.

**Fig. 7:**
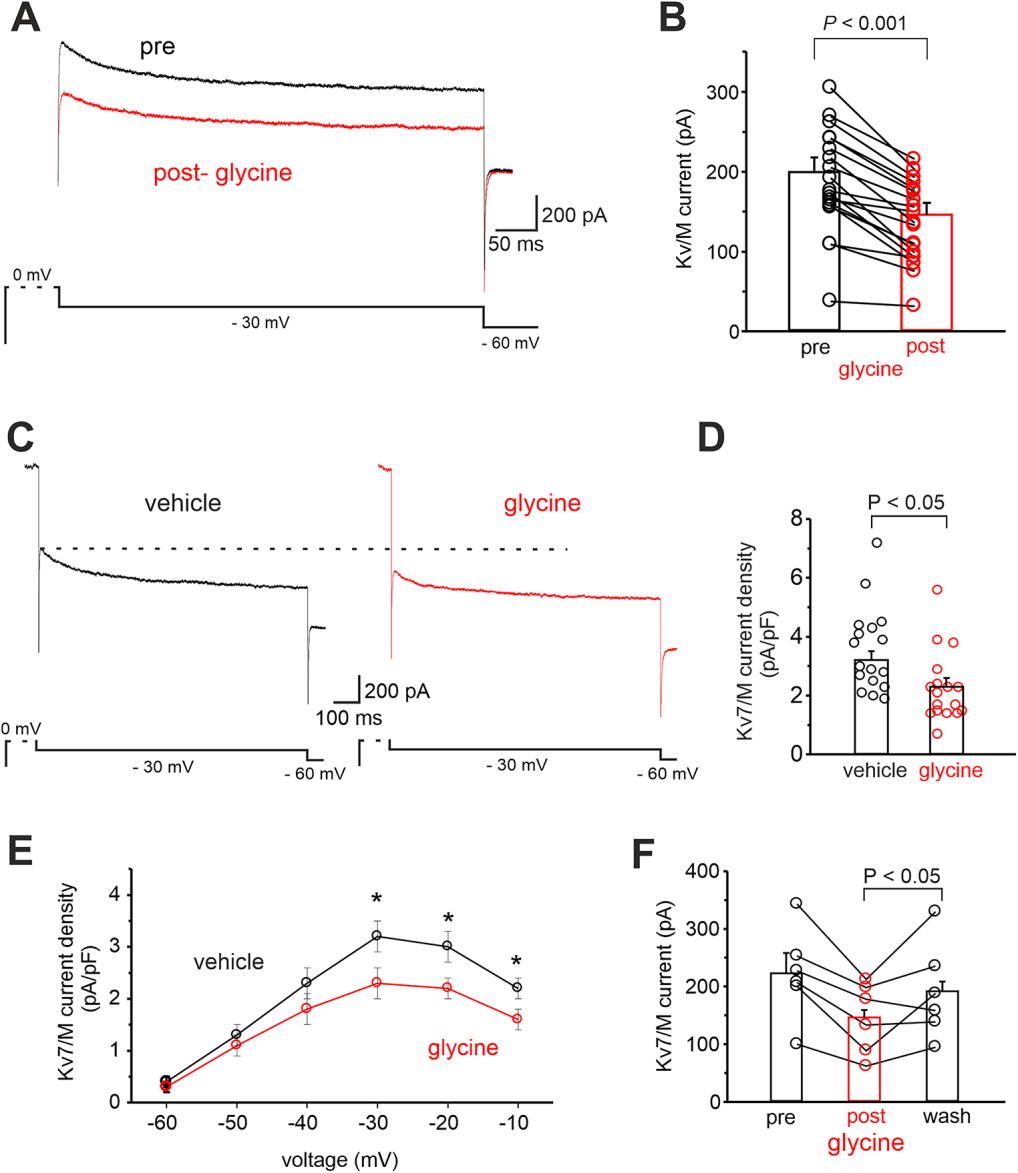
GPR158 signaling pathway activation reduces Kv7/M currents. **A** Representative traces showing Kv7/M currents recorded in MSNs before (black) and after (red) glycine application. **B** Summary plot showing the decreased Kv7/M currents induced by glycine (*n* = 19 cells from 8 mice; *P* < 0.001; paired Student’s *t*-test). **C** Representative traces showing transient Kv7/M currents were recorded in MSNs from NAc slices incubated for 10 min with either glycine or vehicle. **D** Bar graph showing decreased Kv7/M current densities in MSNs in the experimental conditions reported in C. **E** Current-voltage relationship for Kv7/M current densities measured in MSNs from slices incubated with vehicle or glycine (*n* = 17 and 23, respectively; one-way ANOVA followed by Tukey post hoc test; *P < 0.05). These experiments were conducted in the presence of strychnine, picrotoxin, APV and NBQX to isolate the metabotropic action of glycine as for experiments shown n Fig. 1. **F** Bar graph depicting the effect of glycine washout (20–30 min) on Kv7/M currents (*n* = 6 cells from 2 mice; *P* < 0.05; paired Student’s *t-*test)

Under these experimental conditions, we found that Kv7/M current amplitudes were significantly reduced by glycine application (Fig. 7A, B; pre = 199.2 ± 18.4 pA; post = 145.9 ± 15.0; *n* = 20 cells from 7 mice; *P* < 0.0001; paired Student’s *t-*test). Of note, when glycine was applied to MSNs that had been incubated with PKI (1 µM for at least 10 min), no changes in Kv7/M current amplitudes were detected (data not shown; pre = 127.6 ± 18.5 pA; post = 151.4 ± 23.7; *n* = 7 cells from 3 mice; *P* > 0.05; paired Student’s *t-*test), indicating that GPR158-induced negative modulation of Kv7/M current, similarly to the positive modulation of evoked firing, requires PKA-signaling.

We also compared the magnitude of Kv7/M current density in control slices and in slices incubated (for 5–10 min) with glycine. We speculated that activation of the GPR158 signaling pathway would result in a decreased Kv7/M current density in glycine-treated slices.

In support of this hypothesis, we found lower Kv7/M-current density in glycine-treated slices (Fig. 7C, E). Currents density recorded at -30 mV was 3.2 ± 0.3 pA/pF in vehicle-treated slices (*n* = 23 from 8 mice) and 2.3 ± 0.3 pA/pF in glycine-treated slices (*n* = 17 from 6 mice; *P* < 0.05; paired Student’s *t*-test). Collectively, these results suggest that signaling downstream GPR158 activation inhibits Kv7/M-currents in MSNs.

**Fig. 8:**
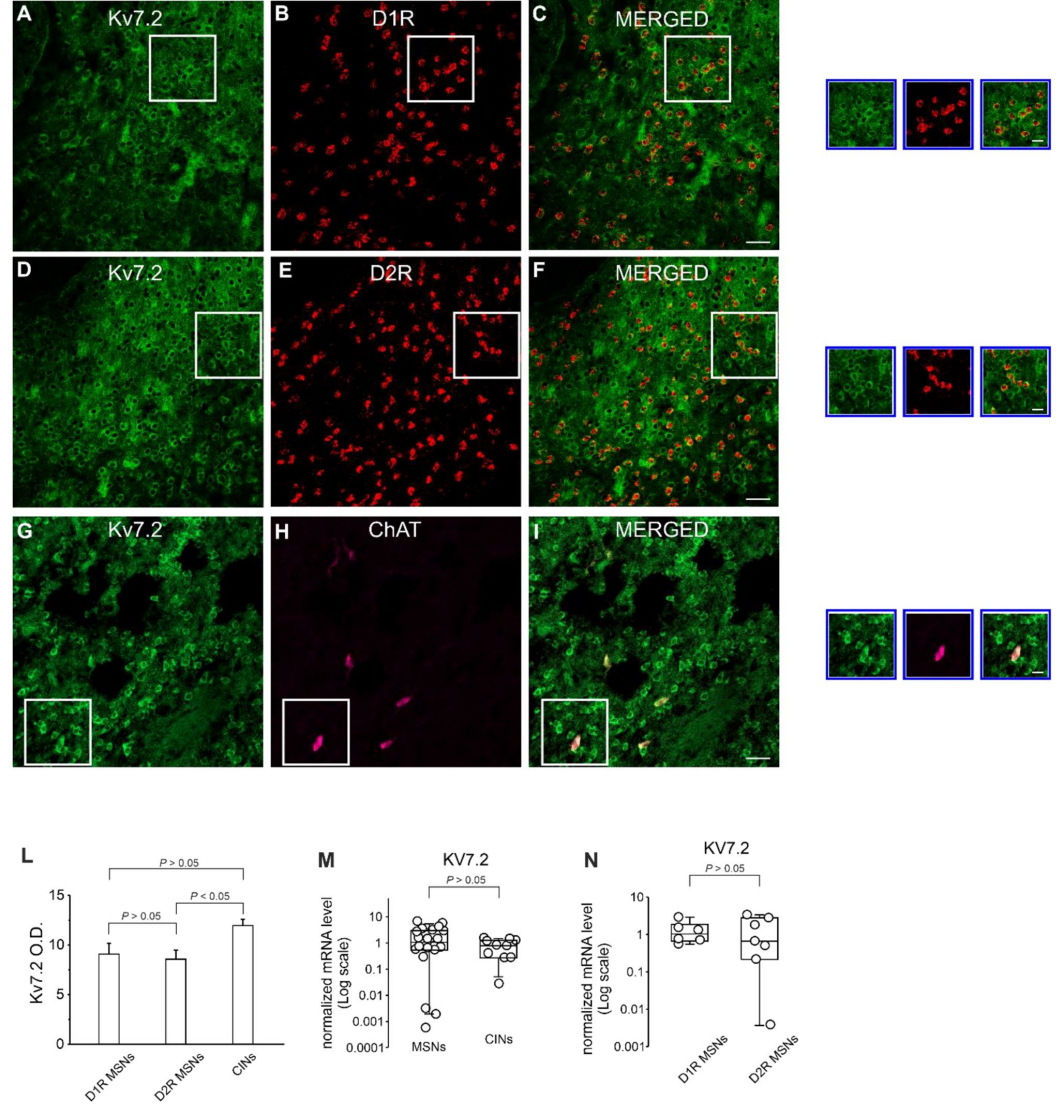
Kv7.2 immunostaining in the NAc and single MSNs expressing D1Rs or D2Rs. Representative RNAscope and immunofluorescence images illustrating high density of Kv7.2 protein signals in NAc D1 MSNs **A**, **C**), D2 MSNs **D**, **F** and CINs **G**, **I**. Noteworthy, protein levels for Kv7.2 channels were not significantly different in D1 MSNs, D2 MSNs, and CINs, as shown in panel **L** summarizing averages of Kv7.2 optical densities. Quantification was done on 50 cells per mice for MSNs and on 15 cells for CINs (*n* = 4 mice). **M** Quantification of Kv7.2 mRNA levels by droplets digital PCR (ddPCR) in single MSNs and CINs. **N** Summary plot showing quantification of Kv7.2 mRNA levels in single MSNs expressing D1Rs or D2Rs. Note that the levels of Kv7.2 mRNA are comparable in both types of MSN (D1R MSNs; *n* = 6 from 2 mice; D2R MSNs; *n* = 7 from 3 mice) and between MSNs and CINs (MSNs: *n* = 20 from 5 mice; CINs: *n* = 11 from 3 mice). Scale bars = 25 μm; insets 10 μm

In the NAc, Kv7 channels are mainly composed of KCNQ2 (Kv7.2) subunits [65–67]. To examine the functional expression of Kv7.2 subunits in NAc MSNs and CINs we first employed RNAscope combined with IF as for experiments shown in Fig. 2. Brain sections containing the NAc were used for multiplexed RNAscope plus IF using Kv7.2 antibody and mRNA probes for D1R, D2R, and/or for double immunofluorescence Kv7.2 plus ChAT. Consistent with our electrophysiological data, Kv7.2 immunostaining was found in MSNs expressing D1R or D2R and in CINs (Fig. 8). Quantitative analysis showed that Kv7.2 signal was quite similar in all cell populations considered (D1R or D2R MSNs; CINs) (average optic densities: D1 = 9.06 ± 0.94; D2 = 8.56 ± 0.79; one-way ANOVA; F _(2,9)_ = 0.07; *P* > 0.05; followed by Bonferroni *post hoc* test) and CINs (average optic density: CINs = 11.95 ± 0.55; one-way ANOVA; F_(2,9)_ = 0.07; *P* > 0.05; followed by Bonferroni *post hoc* test) (Fig. 8A, L). We also evaluated Kv7.2 mRNA levels in single MSNs and CINs by using single-cell qRT-ddPCR. In line with smRNA-FISH results, mRNA levels of Kv7.2 were comparable in single MSNs (D1R or D2R MSNs) and CINs (Fig. 8M, N).

**Fig. 9:**
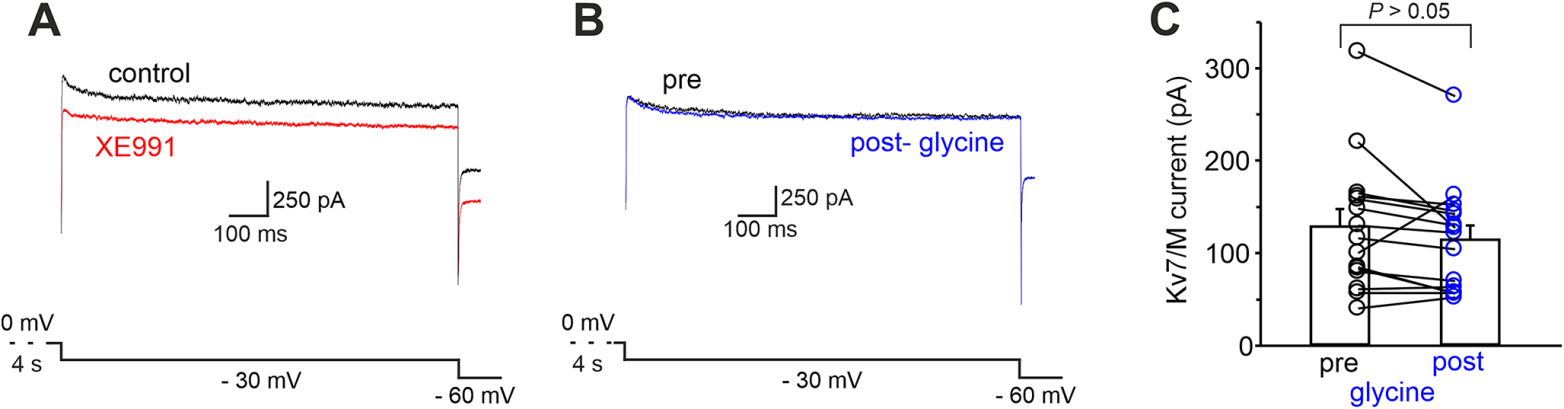
GPR158 signaling pathway activation failed to affect Kv7 currents in NAc cholinergic interneurons. **A** Representative traces showing Kv7 currents recorded at -30 mV before and after XE991 application in a CIN. **B** Representative traces showing Kv7 currents recorded in a CIN before (black) and after (red) glycine application. **C** Summary plot showing that glycine application did not affect Kv7 currents (*n* = 19 cells from 8 mice; *P* > 0.05; paired Student’s *t*-test) in CINs. Recordings were obtained in the presence of strychnine, picrotoxin, APV and NBQX

We next performed whole-cell patch-clamp recordings in voltage-clamp configuration and compared Kv7/M-currents before and after glycine application in CINs. In agreement with electrophysiological data shown in Fig. 1D, E, Kv7/M-current amplitudes were not significantly affected by glycine application (pre = 128.2 ± 19.3 pA; post = 113.9 ± 15.8; *n* = 15 cells from 6 mice; *P* > 0.05; paired Student’s *t*-test; Fig. 9A, C;) thus indicating that GPR158 signaling is not involved in the modulation of Kv7/M-currents of CINs.

### GPR158-dependent modulation of evoked firing and Kv7/M current requires ERK phosphorylation

We then asked how GPR158-PKA signaling decreases Kv7/M-channel in MSNs. It is well known that increased PKA levels in D1-MSNs lead to phosphorylation of ionotropic glutamate receptors (NR2B subunit of NMDAR and GluR1 subunit of AMPAR), DARPP-32, and CREB [48]. More recently, PKA has been shown to phosphorylate many novel substrates including Rasgrp2. Phosphorylation of Rasgrp2 leads to Rap1 activation, followed by recruitment of the (MAPK)1/3 pathway (also known as ERK1/2), which increases the excitability of D1R-MSNs [47]. Interestingly, ERK1/2 directly phosphorylates Kv7.2 at Ser-414 and Ser-476 in vitro, resulting in downregulation of Kv7.2 function [66].

**Fig. 10:**
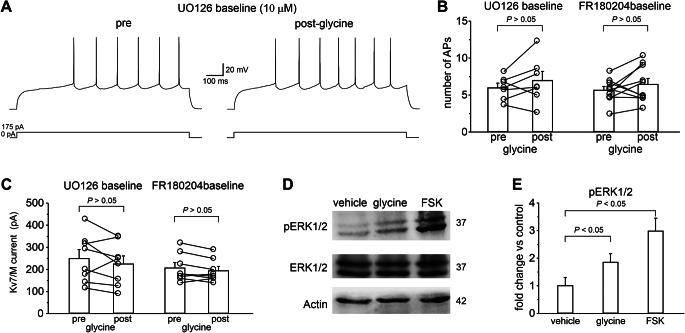
Involvement of ERK1/2 pathway in GPR158-dependent increase of MSN intrinsic excitability. **A** Representative traces showing that when the ERK pathway was disrupted by slices pre-incubation with U0126 (20 min; 10 µM), a specific MEK1/2 inhibitor, modulation of MSN excitability by GPR158 activation was suppressed. **B** Summary plot illustrating quantification of the evoked firing in the experimental conditions reported in A (UO126: *n* = 7 cells from 3 mice; *P* > 0.05; paired Student’s *t*-test) and in an experimental condition in which the ERK pathway was disrupted by pre-incubation of slices with FR180204 (20 min; 20 µM; *n* = 10 cells from 4 mice; *P* > 0.05; paired Student’s *t*-test), a specific ERK inhibitor. **C** Summary plot showing that glycine application did not affect the Kv7.2 currents when U0126 or FR180204 were present in the bath (UO126: *n* = 8 cells from 3 mice; *P* > 0.05; paired Student’s *t* test; FR180204: *n* = 8 cells from 3 mice; *P* > 0.05; paired Student’s *t*-test). **D** Representative western blots of NAc tissue showing increased levels of ERK1/2 phosphorylation in slices incubated with either glycine or forskolin, an adenyl cyclase activator. **E** Densitometry analysis for the blots probed with anti ERK1/2 and anti pERK1/2, normalized to actin (*n* = 3 mice; statistics by ANOVA one way with Tukey’s post hoc test; control vs. glycine *P* < 0.05; control vs. forskolin *P* < 0.05)

Based on these findings, we tested whether ERK1/2 phosphorylation is required for the GPR158-dependent effect on MSN intrinsic excitability. When the ERK pathway was disrupted by pre-incubation of slices with U0126, a specific MEK1/2 inhibitor [66], modulation of MSN firing and Kv7/M-current by glycine were prevented (number of APs: pre = 5.96 ± 0.63; post = 6.93 ± 1.25; *n* = 7 cells from 3 mice; *P* > 0.05; paired Student’s *t*-test; Fig. 10A, B; Kv7/M-current: pre = 249.3 ± 40.6 pA; post = 224.4 ± 36.4; *n* = 8 cells from 3 mice; *P* > 0.05; paired Student’s *t*-test; Fig. 10C). In addition, slice pre-incubation with FR180204, a specific ERK inhibitor, prevented glycine effects on MSN firing and Kv7/M-current (number of APs: pre = 5.59 ± 0.51; post = 6.43 ± 0.81; *n* = 10 cells from 4 mice; *P* > 0.05; paired Student’s *t*-test; Fig. 10A, B; Kv7/M-current: pre = 206.7 ± 23.9 pA; post = 193.7 ± 19.4; *n* = 8 cells from 3 mice; *P* > 0.05; paired Student’s *t*-test; Fig. 10C). We then examined whether incubation of NAc slices with glycine increases the levels of phosphorylated ERK1/2. Consistent with our hypothesis, ERK1/2 phosphorylation increased when GPR158 was activated in NAc slices by incubation (5–10 min) with glycine (Fig. 10D; *n* = 3 mice; statistics by ANOVA with Bonferroni post-hoc test; *P* = 0.013). An increase in ERK1/2 phosphorylation also occurred when NAc slices were incubated with forskolin, a well-known activator of adenylate cyclase/PKA/ERK signaling (5 µM; Fig. 10D, E; *n* = 3, statistics by ANOVA with Bonferroni post-hoc test; *P* < 0.05). Since anti-Kv7.2 phospho-Ser-414 and anti-Kv7.2 phospho-Ser476 antibodies are not commercially available we were unable to confirm whether, in our experimental conditions, the GPR158/PKA/ERK signaling cascade indeed leads to increased Kv7.2 phosphorylation at these sites, as for D1R/PKA/ERK-dependent mechanism [66]. However, by immunoprecipitation experiments, we observed a significant increase in total phosphorylation levels of serine residues (pSER) of Kv7.2 protein following NAc slices incubation (5–10 min) with glycine (Supplementary Fig. 4). Taken together, these results suggest that GPR158 activation, in NAc MSNs, leads to Kv7.2 inhibitory modulation via PKA/ERK cascade.

## Discussion

The present work demonstrates that glycine-dependent activation of GPR158 increases MSN excitability in the NAc. Our data also indicate that this effect is mediated by downregulation of Kv7.2 channels via PKA/ERK-dependent phosphorylation. Notably this novel modulatory mechanism is absent in NAc CINs, likely because of low GPR158 density. Our conclusions are supported by the following experimental evidence: i) activation of GPR158 by glycine results in increased evoked firing and Kv7/M current down-regulation; (ii) the increased GPR158-induced firing is prevented by pharmacological inhibition of PKA signaling; (iii) pharmacological blockade of Kv7/M currents mimics and occludes the GPR158-induced effect on MSN excitability; (*iv*) activation of GPR158 by glycine increases phosphorylation levels of both ERK and total serine residues of Kv7.2; and (*v*) GPR158-induced increased firing and decreased Kv7/M current are abolished by ERK pathway inhibition.

Noteworthy, these glycine’s effects in MSNs have been observed in experimental conditions in which ionotropic GlyRs and synaptic drives have been antagonized by pharmacological blockade. Indeed, most of the experiments were conducted in slices pre-incubated with picrotoxin, strychnine, NBQX, and APV. Since the metabotropic effect of glycine we observed is an excitatory one, contrasting with the largely inhibitory influence of ionotropic GlyR receptors, our findings strongly support the notion that glycinergic transmission in CNS involves interplay between ionotropic and metabotropic systems as for other neurotransmitters.

GPR158 is extensively expressed in CNS, particularly in the striatum and prefrontal cortex [14, 15, 21, 22]. Our use of multiplex RNAscope combined with immunofluorescence and single-cell qRT-ddPCR provided direct evidence of GPR158 expression in the NAc. Furthermore, our data indicate that mRNA and protein levels of GPR158 are comparable in D1R-MSNs and D2R-MSNs (Fig. 2 and Supplementary Fig. 2). Based on these findings and considering that GPR158 stimulation increased the excitability in the majority of recorded MSNs, we speculate that activation of GPR158 enhances the intrinsic excitability of both MSN subtypes.

In our experimental conditions, GPR158 activation failed to significantly affect evoked firing and Kv7/M current in CINs. Our multiplex RNAscope analysis demonstrated significantly lower Gpr158 mRNA and protein expression in CINs compared to MSNs (Fig. 2 and Supplementary Fig. 2). Although we cannot rule out the possibility that GPR158 may activate alternative intracellular signaling cascade in CINs, we speculate that the lack of effect we observed in CINs could be explained by the lower GPR158 expression levels.

In this study, we also show that M-currents in MSNs are downregulated following GPR158 activation. In striatal MSNs, M-currents were primarily attributable to heteromeric Kv7.2/Kv7.3 channels [65–68]. Our RNAscope and single-cell ddPCr experiments confirmed the expression of the Kv7.2 channel subunit, whose activity is modulated by GPR158 signaling only in MSNs.

Operating at sub- and suprathreshold voltages, Kv7/M channels produce an outward potassium current that lacks inactivation and activates near resting membrane potential with slow gating kinetics [52, 69]. Thus, the outcome of M-current is the control of numerous aspects of neuronal excitability including setting membrane potential [70, 71], and afterhyperpolarization [56, 57, 72], interspike interval [73, 74] and theta resonance [62, 75]. Noteworthy, Kv7.2 channels contribute to AHP and AP duration [56, 57]. Neurons with decreased Kv7.2 channels expression and/or activity would have shorter AHP amplitudes and AP broadening, which are features allowing neurons to have higher AP firing frequency. Consistently, in our study these effects were found in MSNs after GPR158 activation and following pharmacological blockade of Kv7/M currents. Furthermore, our finding that GPR158 activation reduces Kv7/M current in MSNs, together with the evidence that the pharmacological blockade of Kv7/M current occludes the GPR158-induced increase in MSN excitability, strongly supports the conclusion that Kv7.2 channel is a downstream target of GPR158 signaling in NAc MSNs.

The Kv7/M-current is transiently reduced by a variety of neurotransmitters that activate Gq-protein coupled receptors [69, 76]. The stimulation of the M1/M3 muscarinic receptors [61, 77], metabotropic glutamate receptors [78], κ and δ opioid receptors [79], AT1 angiotensin receptors [80], purinergic P2Y receptors [81], 5-HT2 serotonin receptors [82], and other stimuli have all been shown to suppress the Kv7/M-current. Gq/11 coupled receptors result in the hydrolysis of PIP2 into diacylglycerol (DAG) and inositol triphosphate (IP3), leading to the activation of PKC which phosphorylates Kv7.2 subunit. The phosphorylation of Kv7.2 at S541 located at the distal segment of the CaM-binding site induces the dissociation of CaM from the Kv7.2 channel. Calmodulin‐deficient KCNQ2 channel has a lower affinity towards PIP2. Therefore, the impaired ability to interact with PIP2 leads to a collapse of the channel pore [83] and profound suppression of the M‐channel activity [84]. Alongside this well-characterized Gq-protein-dependent mechanism, it has been recently shown that Kv7.2 channels can be phosphorylated by PKA/Rap1/ERK pathway [47, 66]. Dopamine increases D1R-expressing MSN excitability and firing rates in NAc via the PKA/Rap1/ERK pathway to promote reward behavior [47]. Furthermore, the D1R agonist inhibited Kv7.2-dependent current and increased D1R-MSN firing rates in the NAc slices, and these effects were abolished by ERK inhibition. Notably, decreased Kv7.2 channel activity was mediated by ERK phosphorylation of Kv7.2 at Ser-414 and Ser-476 downstream of the dopamine/PKA/Rap1 pathway [66].

In line with the above evidence, we found that activation of GPR158 signaling by glycine increased ERK phosphorylation. Moreover, GPR158-induced increased firing and decreased Kv7-currents were abolished in MSNs by ERK inhibition. The anti-Kv7.2 phospho-Ser-414 and anti-Kv7.2 phospho-Ser-476 antibodies are not commercially available and, therefore, it remains to be verified whether activation of GPR158 signaling results in increased Kv7.2 channel phosphorylation levels at these inhibitory phosphorylation sites.

It has been shown that GPR158 regulates the intrinsic excitability of pyramidal cells in superficial layers (L2/3) of the medium prefrontal cortex (mPFC) (Song et al. 2019). This effect was not observed in L5 neurons, and it is mediated by Kv4.2 channel modulation via a cAMP-dependent mechanism. Although GPR158 likely regulates several effectors, our observations suggest that its effect on NAc MSN excitability is mainly mediated by Kv7 channels, which are well known for their role in regulating neuronal excitability (Brown & Passmore 2009).

Our findings led us to propose a mechanism via which glycine regulates neuronal excitability as follows: when glycine acts on MSNs, the PKA pathway is activated to induce Kv7.2 phosphorylation via ERK. Phosphorylation of Kv7.2 decreases the channel activity, which in turn increases cell excitability. Alongside increased excitability induced by changes in AP properties (i.e., AHP, AP half-width), the GPR158/PKA/ERK-dependent modulation of Kv7.2 may affect the transition and duration of up-states in vivo. In the NAc, MSNs exhibit up- and down-states in which the membrane potential oscillates between approximately − 85 and − 65 mV [85] in response to cortical and thalamic glutamatergic synaptic activity. The up-state event is critical to striatal signaling because MSNs generate APs only during up-states. These up-state transitions are of variable duration, sometimes lasting seconds [85, 86]. Previous work has revealed that K^+^ channels are critical determinants of the up-state membrane potential [85]. Kv7.2 channels are suitable for this role because of their ability to open and remain active within the membrane potential range of the upstate. Therefore, negative modulation of Kv7.2 channels by GPR158 activation would increase the responsiveness of MSNs to excitatory cortical and thalamic synaptic inputs. Conversely, decreased activation of GPR158 would reduce the excitability of MSNs during up-states.

In which physiological conditions endogenous glycine may modulate MSN excitability through GPR158 activation? In the adult CNS, glycinergic neurotransmission is most abundant in the spinal cord and brainstem where glycinergic synapses have a well-established role in the control of locomotion and pain processing [2, 87, 88]. Glycinergic innervation is also present in the cerebellum and at low levels in specific regions of the forebrain, where glycine contributes to neuronal inhibition in combination with GABA to maintain the balance between excitation and inhibition [87]. Interestingly, in different brain areas neurons express functional glycine receptors and transporters without the apposition of glycinergic fiber terminals [2, 87]. Therefore, a model emerges by which GPR158 operates through glycine volume transmission. Volume transmission in the brain refers to actions of neuroactive molecules at a distance well beyond the site of release from synapses or cells [89, 90]. Volume transmission offers a communication modality that is more suitable to modulatory and tuning functions because of its slower transmission speed and broader anatomical reach, in comparison to synaptic (wired) transmission, which is specialized for accurate and fast communication. MSN excitability would be modulated by the level of tonic GPR158 activity, which would be determined by the levels of glycine in the extracellular environment. With their ability to control the local glycine availability through glycine transporters [91], astrocytes are ideally positioned to tune GPR158 function. In GPR158-expressing regions with dense glycinergic innervation, such as the brainstem and spinal cord [87, 92], an alternative scenario is conceivable whereby GPR158 operates in a phasic manner by responding to fast synaptic glycine transients. Further studies are required to assess whether GPR158 operates under both a phasic and tonic regime in different brain regions.

Regarding the potential implications of our findings within the context of the pathophysiological role of GPR158 and the glycinergic system in the CNS, it is noteworthy that GPR158 has been demonstrated to be implicated in the etiology of affective disorders, such as cognitive disease and memory loss [12–14]. Interestingly, GPR158 has also been identified as a key mediator of stress-induced depression both in mouse models and in humans [13, 14, 18]. A global ablation of GPR158 led to an anti-depressive phenotype in mice, characterized by a lower susceptibility to learned helplessness and reduced anhedonia [13, 14]. Notably, such behavior was rescued by the viral overexpression of GPR158 in the mPFC [14]. Moreover, in multiple behavioral paradigms including the sucrose preference test for anhedonia, GPR158 KO mice showed resilience to chronic stress-induced depression [14]. Since the NAc is a pivotal node in the limbic basal ganglia loop, and its dysfunction results in psychiatric diseases including stress-related disorders such as depression and anxiety (Francis & Lobo, 2017; Gunaydin & Kreitzer, 2016), it is tempting to speculate that Glycine/GPR158-dependent enhancement of MSN excitability we observed could conceivably represent a mechanism by which elevated extracellular glycine levels may promote stress-dependent decisional strategies. Therefore, our findings encourage further studies aimed at elucidating the role of NAc glycine and GPR158 in stress-associated physiological states.

### Electronic supplementary material

Below is the link to the electronic supplementary material.

Supplementary Material 1

## Data Availability

The datasets generated and/or analyzed during the current study are available from the corresponding author upon reasonable request.
