# Supplementary material for: Glycine-induced activation of GPR158 increases the intrinsic excitability of medium spiny neurons in the nucleus accumbens
Source: Cell Mol Life Sci. 2024 Jun 17;81(1):268. doi: 10.1007/s00018-024-05260-w (PMC11335193; doi:10.1007/s00018-024-05260-w)
Supplement: Supplementary file 1 — Supplementary Material 1 [file 18_2024_5260_MOESM1_ESM.docx]

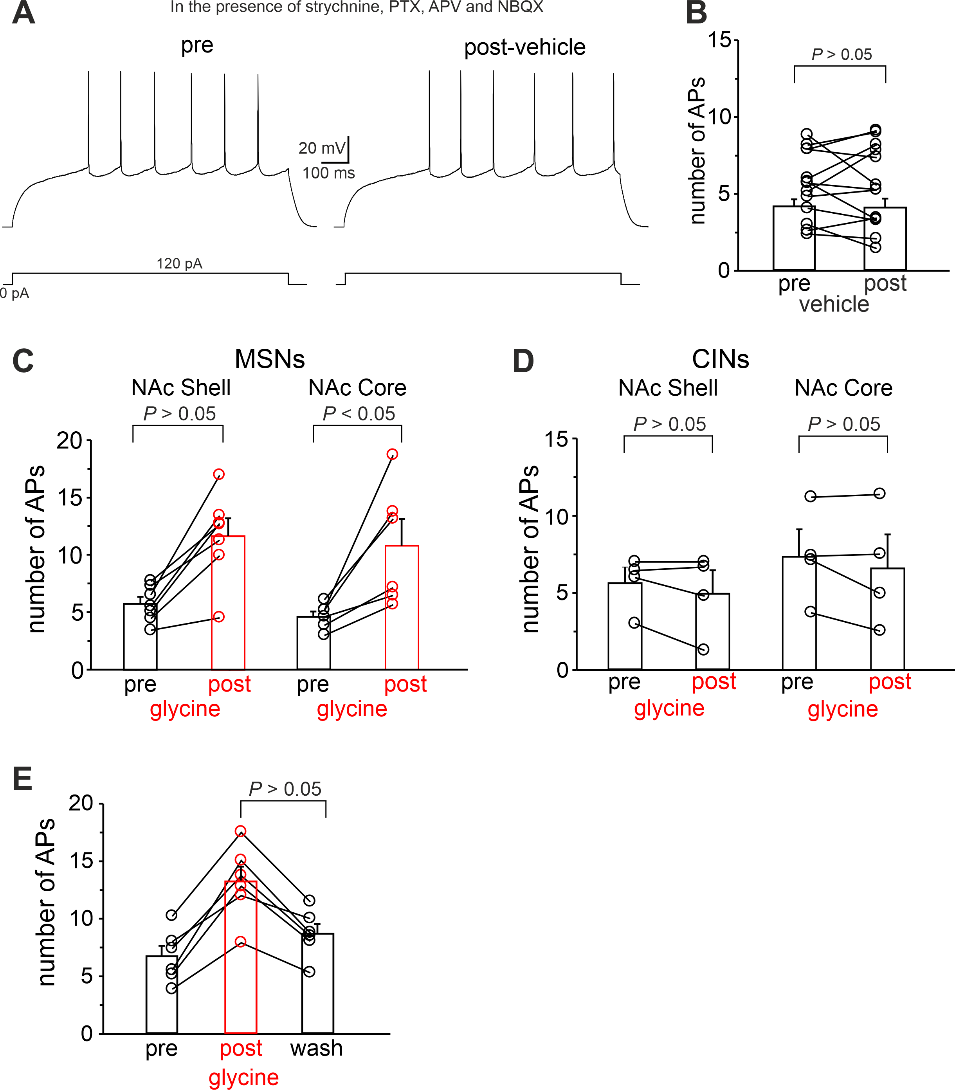


**Supplementary Fig. 1** Glycine’s effect on evoked firing was independent of MSN localization in the NAc core and shell. **A** Representative traces and summary plot **B** illustrating that vehicle application did not affect evoked firing in MSNs (n = 13 from 6 mice; *P* > 0.05; paired Student’s *t-*test). **C** Bar graph depicting quantification of the glycine-induced effect of evoked firing in MSNs of NAc shell and core. **D** Summary plot illustrating that glycine application did not affect evoked firing in CINs of both NAc shell and core. **E** Bar graph depicting the effect of glycine washout (20-30 min) on MSN evoked firing (n = 6 from 2 mice; *P* > 0.05; paired Student’s *t-*test).


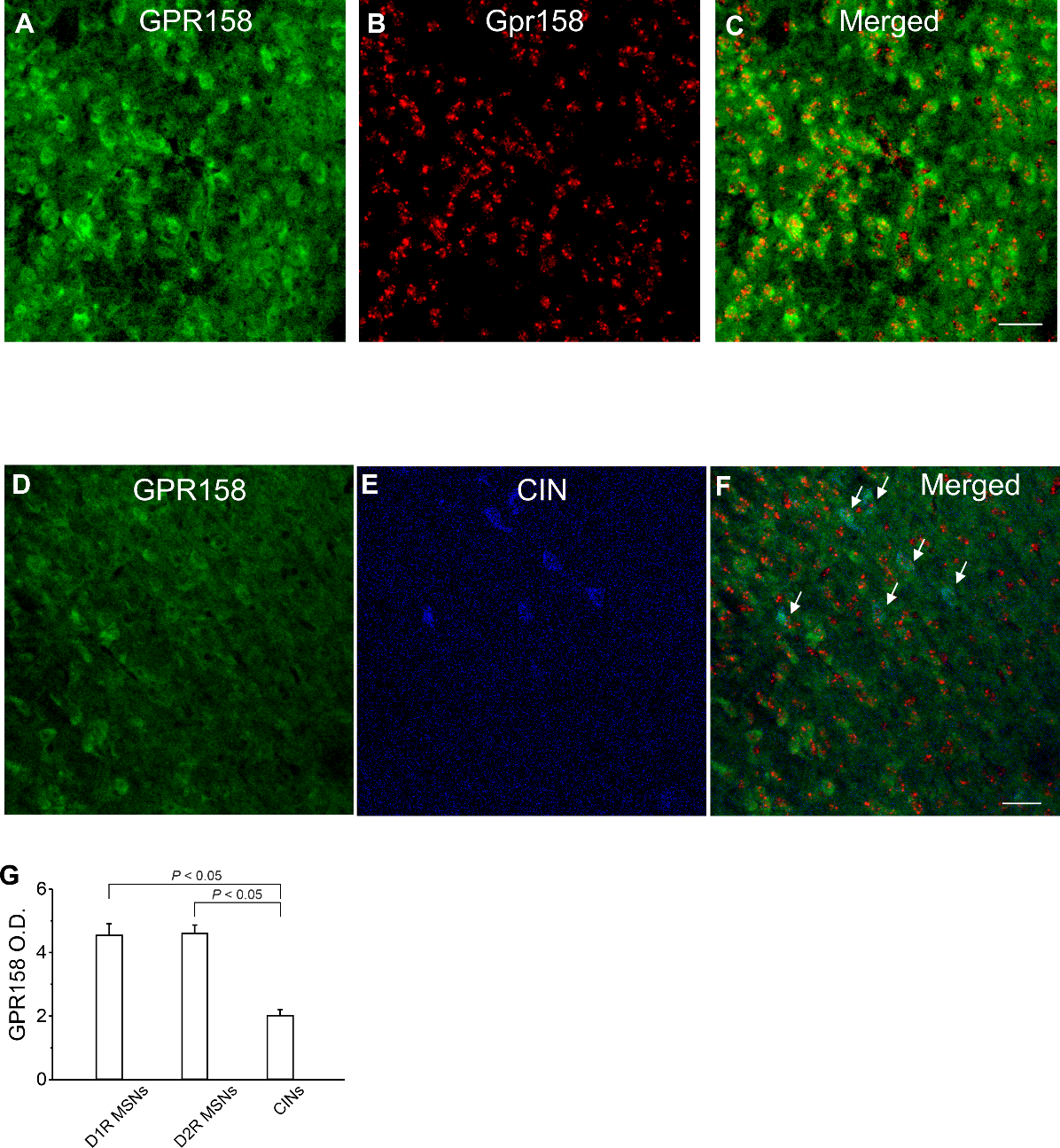


**Supplementary Fig. 2** Different protein levels of GPR158 in MSNs and CINs. **A, C** Representative confocal images showing a high degree of colocalization (94%) between the GPR158 antibody (protein; A) and Gpr158 mRNA (B). **D, F** Noteworthy, GPR158 protein levels were lower in CINs compared to MSNs as shown in panel **G** summarizing averages optical densities of protein levels in MSNs and CINs, considering the cellular surface (Area). Quantification was done on four sections/mice (n = 4 mice). Scale bars = 25 µm; insets 10 µm.

**
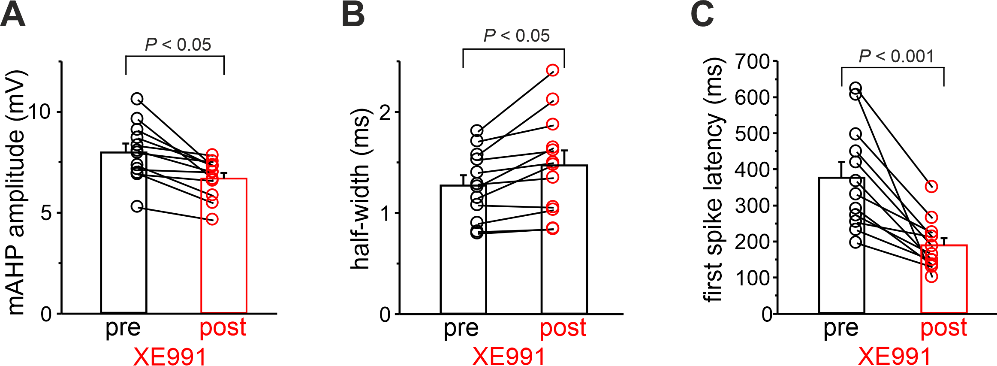
**

**Supplementary Fig. 3** XE991 application mimicked the effects of glycine on the first spike latency, mAHP, and half-width. **A, C** Summary plots showing the mean values of mAHP, AP half-width, and first spike latency before and after XE991 application (n = 12 cells from 5 mice).

**
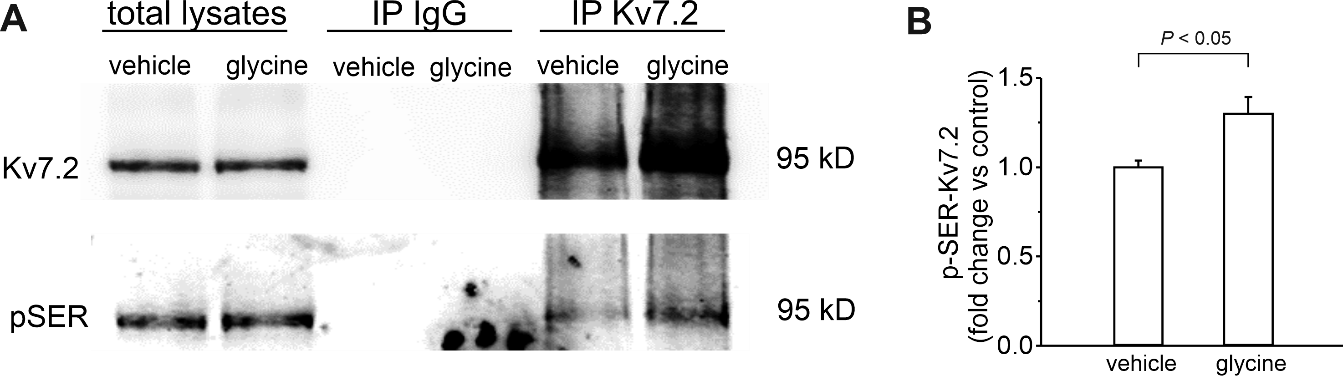
**

**Supplementary Fig. 4** Glycine-dependent GPR158 activation increases the total phosphorylation of serine residues of Kv7.2. **A** Representative immunoblots of NAc tissue showing increased levels of Kv7.2 phosphorylation (total pSER) in slices incubated with glycine, assessed by immunoprecipitation. **B** Densitometry analysis showing the ratio between pSER signal and Kv7.2 levels (n = 3 mice; statistics by ANOVA one way with Tukey’s post hoc test; *P* < 0.05).
